# Can Evaluative Conditioning Change Well-Established Attitudes Towards Popular Brands? Your Brain Says Yes Even Though Your Mouth Says No

**DOI:** 10.3390/brainsci9050106

**Published:** 2019-05-10

**Authors:** Shannon Bosshard, Monika Koller, Peter Walla

**Affiliations:** 1 School of Psychology, University of Newcastle, Newcastle, Callaghan NSW 2308, Australia; shannon.bosshard@uon.edu.au; 2 Department Marketing, Vienna University of Economics and Business, 1020 Vienna, Austria; monika.koller@wu.ac.at; 3 CanBeLab, Department of Psychology, Webster Vienna Private University, 1020 Vienna, Austria

**Keywords:** brand attitude, brain imaging, triangulation, neuroconsulting

## Abstract

In the present study, using both implicit and explicit measures, we addressed the issue of whether strongly developed relationships towards brands could be modified through the use of evaluative conditioning. Using an online survey, individual participant brand lists were created, and formed the basis of this experiment. Participants were then exposed to conditioning during a longitudinal study. Throughout the experiment, a combination of explicit and implicit measures was used to assess changes in attitude. Specifically, participants were asked to rate the brand names on a Likert-type scale. Simultaneously, changes in the brains electrical activity in response to the brands were recorded via electroencephalography (EEG). Upon completion of this task, participants underwent two Implicit Association Tests (IAT; one for liked brands and one for disliked brands). There were two main findings of this study. Firstly, no significant changes in attitude were observed via the use of explicit measures, and those that were found relating to the IAT were regarded as questionable. Secondly, EEG presented consistent results which showed that conditioning elicited changes in cortical activity towards both liked and disliked brands, which suggest it may be a useful tool in measuring the impact of evaluative conditioning that is not reflected in verbal responses.

## 1. Introduction

For the majority of existing businesses, progressive growth is stimulated by the successful marketing of products and/or services. Consumers are driven to purchase brands or products they like, and avoid those they don’t. As a result, marketers are constantly looking to modify consumer’s attitudes towards their brands or products. Currently, the most utilised method of changing consumer attitudes is evaluative conditioning [1]. This process involves the repeated pairing of a conditioned stimulus (CS; e.g., a brand name) with an unconditioned stimulus (US; e.g., affective sound or picture). Eventually, the repeated pairings result in a transfer of affect from the US to the CS. A well-known example of evaluative conditioning in advertising, are the “Just Do It” commercials run by Nike. Throughout each commercial, the Nike brand name (CS) is repeatedly presented together with images of individuals working hard to achieve greatness (US). It is assumed that purchasing Nike products will allow the consumer to reach their potential more easily and more comfortably. As a result, liking of the brand is thought to increase. Although research suggests that evaluative conditioning can lead to an increase in brand awareness [2,3], sales [4,5], and brand positivity [6], there is debate as to whether it can reliably change well established attitudes towards familiar brands [6,7].

Marketing literature constantly stresses the importance of brand identity on the consumer’s perception and decision making process [8]. Extant research shows that consumers use brand names as an indicator of performance and quality [9]. Such literature even goes so far as to posit that brand perceptions can influence sensory processing (e.g., taste) [10,11]. Findings such as these stress the importance of branding. In a market where competitors can easily copy product characteristics, it is essential that a strong brand identity and personality are formed in order to build brand equity [12]. Understanding the importance of a strong brand, many companies have implemented conditioning paradigms in attempts to increase positive brand perceptions. The most common forms of conditioning involve businesses using heuristic stimuli such as music, celebrities, happy and fun scenes, and bright colors in order to persuade potential consumers that the product being offered is beneficial. 

According to Stammerjohan et al. [13], highly familiar brands are characterized by well-established, relatively stable attitudes that are somewhat resistant to the effects of advertising. In the majority of cases, research suggests that changing attitudes towards mature brands is unsuccessful [14,15,16]. Latent inhibition (LI) is the process whereby people’s attitudes towards a stimulus are resistant to change as a result of previous exposure to that stimulus. Null findings as a result of latent inhibition are common within marketing literature [7,17,18]. Such findings are intriguing given that brand name attitudes are entirely learned, highly semantic [18], and can be derived and shaped without the individual actually having any direct experience with the brand [19,20]. This paper suggests that many of the abovementioned null findings occur as a result of an overreliance on explicit measures, the preferred option of many marketing firms.

Whilst the majority of marketing research sees the inclusion of explicit measures of attitudes, very few acknowledge the role of implicit processes [21] and thus, fail to utilize implicit measures. Explicit attitudes are said to be contemplative, formulated through reasoning [21], and are usually measured in terms of fully conscious responses such as ticking boxes, pressing buttons, or giving verbal responses. In contrast, implicit attitudes are associations that are automatically activated in the presence of relevant stimuli without any conscious awareness of evaluation [22]. These attitude aspects require more sophisticated methodological approaches (see later). The distinction between these two types of attitudes arose after constant reports suggested that people either cannot, or do not want to fully explain their preferences [23,24].

Attitude research that is seen to compare implicit and explicit attitudes constantly reveal discrepant findings [25,26,27,28]. For instance, Walla et al. found that when eating ice cream, chocolate or yoghurt, although participants had no stated preference for particular food items, implicit responses showed that ice cream was preferred [28]. Similar findings were presented by Grahl et al., who reported that specific bottle shapes can elicit a non-conscious change whilst explicit ratings remain constant [26]. More specifically, although both males and females stated that one particular bottle (out of three) was the least attractive, physiological responses (startle reflex) suggested that this bottle elicited a negative affect in only male brains.

Neuroscience is able to offer an alternative explanation to latent inhibition regarding the discrepant findings between implicit and explicit attitudes. According to Walla, Brenner and Koller [29] contemplation and reasoning inevitably give rise to cognitive pollution. In more simplistic terms, the more conscious/cognitive processing that takes place during the evaluation of a stimulus, the less reliable/more polluted the response becomes, with respect to raw affective processing [30]. Given these discrepancies, it is proposed that cognitive pollution is an alternative explanation to the null findings where latent inhibition has regularly been presented in psychological studies. 

Given all these findings, it is imperative to consider alternative means of assessing brand attitude that complete traditional approaches. As a result, the rationale for the present study is to assess well-established liked and disliked brand attitudes using a multidimensional approach. In doing so, the sensitivity of implicit and explicit measures will be utilized to detect baseline attitude in addition to potential changes in attitudes as a result of evaluative conditioning.

Within consumer contexts, of the implicit measures currently utilized, the Implicit Association Test (IAT) is arguably the most popular measure of consumer attitudes and whilst the use of the IAT has primarily been within psychological studies, its application within consumer contexts is growing [31]. The IAT assumes that there is an associated network whereby concepts of memories, attitudes and valence are all intrinsically linked [32,33]. It is inferred that the faster a participant is able to respond to a stimulus (or stimulus pair), the stronger the implicit association. Whilst the IAT seems to be a promising measure of implicit attitudes, it has been met with substantial criticisms regarding its susceptibility to cognitive processes [34,35]. In sum, such criticisms have raised serious concerns regarding the IATs legitimacy of an exclusively implicit measure of attitudes. Only recently, a four-step model has been published to enhance the IAT [36], which might help future studies, but still the IAT should be used with great caution.

Electroencephalography (EEG) is an emerging technique used to assess the affective components related to implicit attitude. It has been proposed that EEG is capable of distinguishing between positive (approach) and negative (avoidance) affect [37,38]. The asymmetry model proposed by Davidson et al. [39] suggests that greater relative left frontal EEG activity is associated with the processing of positive/approach affects, whereas greater relative right frontal EEG activity is associated with the processing of negative/avoidance affects. Within marketing contexts, Ravaja et al found that a reduction in the price of certain products elicited relatively greater left frontal activation. In addition, the occurrence of greater left frontal activation was associated with an increased likelihood for participants to purchase goods [39].

With regard to EEG, asymmetry effects aren’t the only means of investigating attitudes. Of interest to the present study is one of the most empirically valid EEG approach as an index of motivation and affect, the late positive potential (LPP) [40]. The LPP is a late, positive event related potential (ERP) component that becomes evident at around 300ms following stimulus onset and can exceed 5000ms. The LPP has been extensively used within the literature and as a result, has received psychometric endorsement which revealed that the LPP demonstrated good to excellent reliability as a measure of emotion/affective processing [40]. Stimuli that are seen to be more affective and thus, more motivationally relevant are said to evoke the largest LPPs [40]. In addition, regardless of valence, LPP effects are reported to be greater in the right hemisphere than the left [41].

In the present study a multi-session, evaluative conditioning paradigm (varying numbers of conditioning rounds) was utilized with the aim of modifying participant’s attitudes towards their most liked and disliked brand names (established brand attitudes). Participants were initially presented with well-established brand names and their baseline attitudes were recorded via self-report. Subsequent visits to the laboratory aimed to modify participant’s brand attitudes, and to investigate subsequent changes using both subjective (self-report) and objective measures (IAT and EEG). We hypothesized that the different measures are differently sensitive to evaluative conditioning effects on brand attitude, and thus provide a more detailed understanding of brand attitude in general and of how to best detect conditioning effects in general. 

## 2. Materials and Methods

### 2.1. Participants

Initial recruitment for the study involved 22 participants, two of whom were excluded following pre-assessment of brand attitudes. The mean age of the remaining 20 participants (10 females) was 22.81 (SD = 2.37). All participants were tertiary education students recruited by word of mouth. All participants volunteered and gave written informed consent. Participants were right handed, had normal or corrected to normal vision, were free of central nervous system affecting medications or substances (including alcohol, caffeine and nicotine), and had no history of neuropathology. Participants were financially reimbursed for their time and travel. The study was approved by the University of Newcastle Ethics Committee.

### 2.2. Stimuli

The initial stimulus list for pre-assessment comprised of 300 subjectively chosen common brands names, familiar to people from Australia. Using an online survey, participants provided a subjective rating of like or dislike for each brand name on a 21-point analogue-type scale, ranging from −10 (Strong Dislike) to 10 (Strong Like). In addition, brands that had received a rating of zero by participants formed the list of neutral brands. Of the neutral brands, 80 were used as filler brands (brand which were repeatedly presented, but not conditioned). This allowed us to compare target brand effects with non-conditioned neutral brands, which helped rule out pure brand name repetition effects (see Figures 10 and 11).

#### Conditioning Stimuli

In order to condition the target brand names, auditory stimuli from the International Affective Digitized Sound system (IADS) were used [42]. The IADS consists of 111 sounds of an affective nature and was designed specifically to provide a better control of emotional stimuli relating to sounds. All 111 sounds in this database have been pre-evaluated regarding emotional valence (and arousal), thus allowing the sounds to be matched in terms of their affect (positive or negative). The 30 selected unpleasant sounds had a mean pre-evaluated valence rating of 3.1 (SD = 0.57) and the 30 selected pleasant sounds had a mean valence rating of 6.7 (SD = 0.75). Mean pre-evaluated arousal ratings for both categories was kept the same at 6.6 (only their SDs differed slightly, for negative sounds it was 0.48 and for positive sounds it was 0.41). All sound stimuli were paired randomly with each of the brand names (i.e., evaluative conditioning).

### 2.3. Individual Pre-Assessment of Brand Attitudes 

Participants subjectively rated 300 brand names using an online survey (via www.limesurvey.com), prior to entering the lab. We required participants to view each brand name and indicate their attitude towards it using a mouse/track pad on the provided slider. Participants were explicitly instructed not to adjust the slider if they were unfamiliar with a particular brand. Rating a brand as neutral required the participant to manually click “0”. This phase of the experiment occurred at a time of the participant’s choosing, with choice of computer also left to their discretion. The survey took on average 15–20 min to complete. Participants who demonstrated adequate familiarity and attitude scope were eligible for the experimental phase of the study. That is, participants who had insufficient brand name attitudes to construct a stimulus list with discernable positive and negative target items were excluded from the experiment. Two participants were unable to further participate due to such inadequate brand pre-assessment.

### 2.4. Lab Experiment

**Figure 1:**
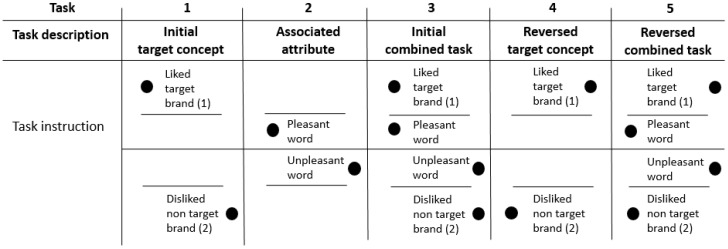
Modified version of the original IAT (adapted from Greenwald et al. [33]. Filled black circles on the left or right of the stimulus correspond to left and right (respectively) button presses. Task 3 = congruent, Task 5 = Incongruent condition. Participants completed this task during each of the lab sessions, once for a liked brand and once for a disliked brand.

Following completion of pre-assessment, we invited eligible participants individually into the lab. During this first session, we collected baseline measurements of explicit and implicit attitudes towards brand names [38]. Explicit measurement involved subjective self-report, whilst implicit measures were collected using electroencephalography (EEG) and the IAT (see Figure 1). Upon entering the lab, participants were seated comfortably in front of a 32” monitor (resolution of 1024 × 768 pixels). We connected participants to a BioSemiActiveTwo EEG system (BioSemi, Amsterdam, The Netherlands) and measured potential changes using 64 cranial electrodes as well as eight external reference electrodes placed lateral-ocularly, supra-ocularly, infra-ocularly, and on the mastoids. 

We used the computer program “Presentation” (NeuroBehavioral Systems, Albany, United States) to visually present the appropriate instructions and individualized stimulus lists. The presentation of stimuli in addition to all psychophysiological signal recording was conducted from a separate room. Participants were given a brief overview of the study during set up of the equipment. We commenced testing with the participant by themselves in a dimly lit room to ensure adequate focus on the stimuli. A white fixation-cross appeared on a black background for 500ms, followed by a brand name for 5s. Participants provided a self-reported rating between 1 (Strong Dislike) and 9 (Strong Like) for each brand using a standard keyboard, whilst it was on screen. Compared to the initial online rating performance, a different rating style was chosen for the actual experiment in order to avoid similarity effects. Brain potential changes and self-report were collected for the 60 target brands. To reduce fatigue effects participants were provided a break halfway through this stage. Overall, it took approximately 30 min to complete. Participants were then asked to complete 5 rounds of the IAT (see Figure 1 for modified IAT and further information below).

Following the completion of the IAT, participants were exposed to one, five and ten rounds of conditioning (total of 16 conditioning rounds; across three separate conditioning trials at three different dates). One round of conditioning lasted approximately six minutes. Participants were allowed to take breaks as required. The duration between lab visits was standardized as best as possible and participants were required to attend subsequent sessions between 2 and five days from their last.

### 2.5. Data Recording and Processing

#### 2.5.1. Explicit Data

Mean self-reported ratings were compared by using paired-sampled *t*-tests. Within participant’s individualized brand lists, participant’s 30 most liked brands were conditioned negatively and their 30 most disliked brands were conditioned positively. 

#### 2.5.2. Implicit Association Test (IAT)

We used a modified version of the original IAT [31], which consisted of 5 separate discrimination tasks each with 30 visual presentations to be classified as either a target or non-target stimulus. The 60 brands rated previously by participants as liked or disliked became the target brands. In task 1 (initial target concept) study participants were asked to discriminate between visual stimuli either related to their individually rated most liked (or disliked) brand (target brand) or related to a neutral brand (non-target brand). Study participants were required to press the “A” key for target brand and the “L” key for non-target brand. In task 2 (associated attribute) participants were visually presented with valenced words and asked to press the “A” key for pleasant words (eg., beautiful, healthy, happy, perfect) and the “L” key for unpleasant words (eg., frighten, angry, sad, worthless). In task 3 (initial combined task) tasks 1 and 2 were combined and formed the congruent condition. Study participants were asked to press the “A” key in case of target brand and pleasant words and the “L” key when presented with a negative word or a non-target brand. Task 4 (reversed target concept) was similar to task 1, however participants were asked to press the “A” key for non-target brands and the “L” key for target brands. Finally, task 5 (reversed combined task; incongruent condition) was a combination of task 2 and task 4. Participants were required to press the “A” key in case of non-target brands and pleasant words and the “L” key when presented with a negative word or a non-target brand. A comparative analysis was made between reaction times of participants during task 3 (congruent condition) and task 5 (incongruent condition). During each of the blocks, stimuli were presented for 300ms; however, participants were given 1500ms to respond during each trial. Between each stimulus, a fixation cross was presented for 300ms and between the fixation cross and the following stimulus, was another 700ms gap.

#### 2.5.3. Event related potentials

We recorded brain potential changes at a rate of 2048 samples/second using a 64-channel BioSemiActiveTwo system and ActiView software (BioSemi, Amsterdam, The Netherlands). Data sets were processed individually using EEG-Display (version 6.3.13; Fulham, Newcastle, Australia). During processing, we reduced the sampling rate to 256 samples/second and applied a band pass filter of 0.03Hz to 30Hz. Blink artefacts were corrected by referring to the supra-ocular external electrode. In particular, eye blink artifacts were corrected using a set of linear regression weights at each EEG electrode derived from an averaged eye blink taken from those electrodes [31]. Further, segments of the EEG record containing gross artifact (like muscle activity) were detected by an automated procedure that applied amplitude thresholds within a number of frequency bands. Trials containing artefact exceeding ±100 μV were excluded. The data was coded to brand type (i.e., liked, disliked and filler). We established epochs from -100ms prior to stimulus onset (a baseline), to 1400ms following stimulus onset. The resultant epochs were baseline corrected and an average was generated across single trials for each condition. The individual data sets were then re-referenced to a common average. We produced averaged ERP figures to broadly assess effects of brand type over the entire epoch of interest. In order to eliminate noise generated by eye movements, we conducted horizontal, vertical and radial eye movement corrections [43].

Subsequent statistical analysis saw epochs divided into 200ms blocks and the mean amplitudes calculated by means of Analysis of Variance (ANOVA). After main effects were reviewed, paired *t*-tests and randomisation tests [44,45] were conducted to assess differences between conditions during each time frame. Given that numerous, recent papers are seen to focus on frontal and parietal sites when investigating attitudes and behaviour [46,47,48,49], we too chose to focus on similar sites. Specifically, frontal electrodes sites AF3 and AF4 were chosen in addition to parietal sites P5 and P6. Following conditioning, each ERP was compared to the session which preceded it.

## 3. Results

### 3.1. Explicit Responses (Self-Report)

**Figure 2:**
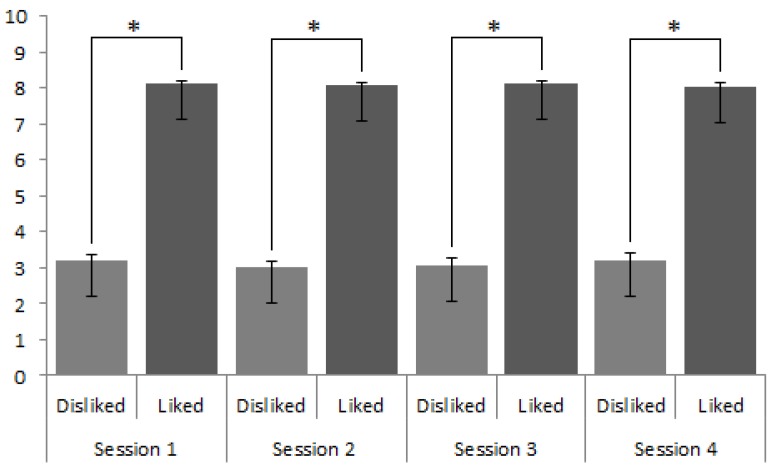
Self-report responses towards liked and disliked brands throughout all four sessions. While liked brands were consistently rated as more positive compared to disliked brands across all sessions (* denotes *p* < 0.001) these ratings did not change between sessions.

For the explicit data, we conducted a 2 (Brand type: liked, disliked) × 4 (Conditioning rounds: zero, one, five, ten) repeated measures ANOVA to determine whether there were any effects on brand rating as a result of conditioning. Results revealed that there was no significant brand type by session interaction (F(3,291) = 0.30, *p* = 0.826, η^2^ = 0.003); See Figure 2).

### 3.2. IAT

For IAT data, in line with previous literature, all incorrect responses as well as any responses falling outside of three standard deviations from the mean were removed [50,51,52]. Two, 2 (Phases: congruent, incongruent) × 4 (Session: one, two, three, four) repeated measures ANOVAs were conducted to assess differences between the congruent and incongruent phases of both liked and disliked brands throughout all sessions of the study. For liked brands, a significant phase by session effect was recorded (F(3,672) = 119.25, *p* < 0.001, η^2^ = 0.347). Similarly, with respect to disliked brands, a significant phase by session effect was recorded (F(3,672) = 176.94, *p* < 0.001, η^2^ = 0.441).

**Figure 3:**
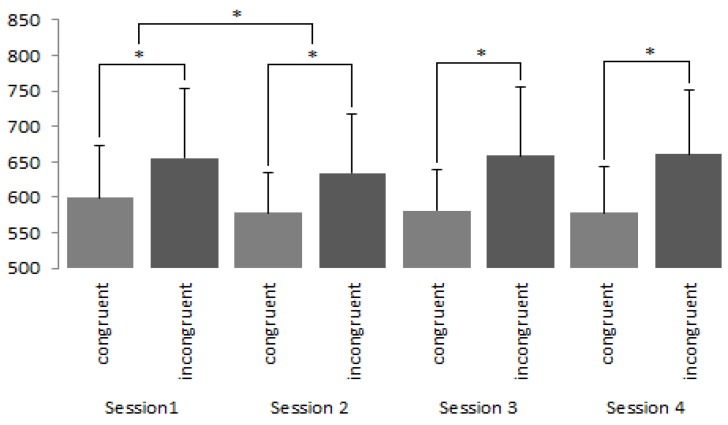
IAT results (in milliseconds) reflecting responses towards liked brands in the congruent and incongruent phases. Whilst significant differences occurred between the congruent and incongruent phases, reaction times were not seen to vary across sessions. * denotes *p* < 0.001.

**Figure 4:**
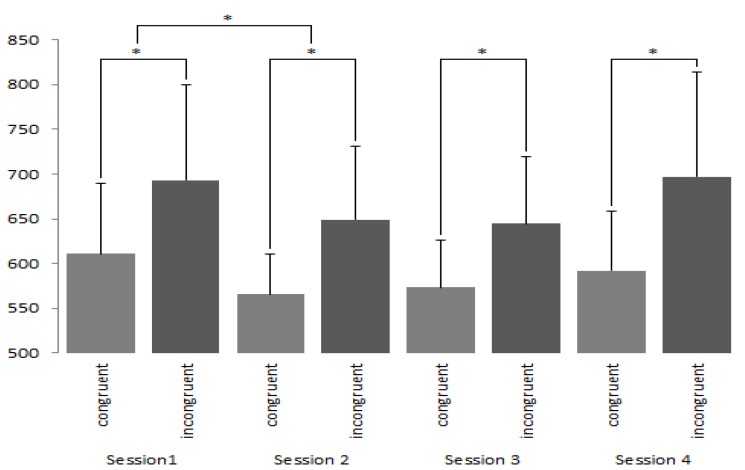
IAT results (in milliseconds) reflecting responses towards disliked brands in the congruent and incongruent phases. Whilst significant differences were witnessed between the congruent and incongruent phases, reaction times were not seen to vary across sessions. * denotes *p* < 0.001.

Following this, *t*-tests were used to compare the congruent and incongruent stimuli during each session for both liked and disliked brands (See Figure 3 and Figure 4). Throughout each session, reaction times between congruent and incongruent conditions were seen to differ significantly from one another for both liked and disliked brands (*p* < 0.001).

Further analysis revealed that the reaction time of participants between sessions one and two for congruent phases were seen to decrease for both the liked (*t* = 8.307; *df* = 224; *p*< 0.001, *d =* 0.17; two tailed) and disliked brands (*t* = 12.777; *df* = 224; *p* < 0.001, *d* = 0.33; two tailed). Similarly, for the incongruent condition, reaction times were also seen to be significantly reduced for both the liked (*t* = 10.667; *df* = 224; *p*< 0.001; *d =* 0.17, two tailed) and disliked brands (*t* = 15.957; *df* = 224; *p*< 0.001, *d* = 0.29; two tailed) between sessions one and two. Finally, for disliked brands, session four saw a significant increase in reaction times, *p* < 0.001. Despite these significant findings we interpret our IAT data with great caution (see discussion section).

### 3.3. Implicit Attitudes - Event Related Potentials

#### 3.3.1. Liked Brands

**Figure 5:**
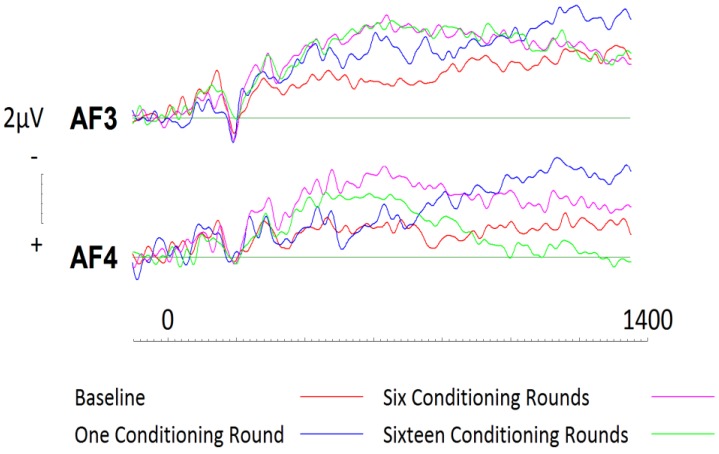
ERPs generated during each conditioning round for liked brands across frontal electrode sites AF3 and AF4. Gradual changes between each session are evident across both electrode sites as brands were conditioned more negatively however, these changes were not significant.

To assess conditioning effects with respect to liked brands, a 2 (hemisphere: left, right) × 4 (Session: one, two, three, four) repeated measures ANOVA was conducted. No significant effects were present across frontal electrode sites AF3 or AF4 (see Figure 5). Following visual inspection of the ERP curves, a Monte Carlo permutation-based analysis (randomization test), as described by Maris and Oostenveld [45], was conducted (See [44] for method). Randomisation tests are considered to be an equally conservative (nonparametric) approach when compared to traditional approaches however, there is no reliance on having normalized data. Epochs were processed in 200ms blocks beginning at 400ms. Randomisation tests (based on 1000 permutations) at electrode site AF3 revealed that activity elicited at baseline was significantly more positive than activity elicited during subsequent sessions. This effect was seen to continue until approximately 1200ms after stimulus onset, and was most significant between 800–1000ms (*r* = 0.64 (*p* = 0.005; two tailed). Subsequent analyses were undertaken at site AF4. Again, liked brands at baseline were seen to elicit significantly more positive activity than the following three sessions, however, this effect was only observed between 400 and 600ms (*r* = 0.48 (*p* = 0.028; two tailed).

**Figure 6:**
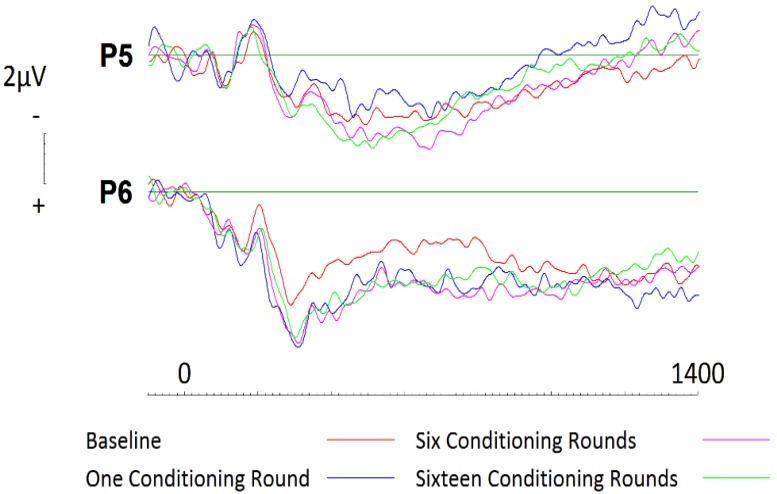
ERPs generated during each conditioning round for liked brands across parietal electrode sites P5 and P6. A significant increase in activity is apparent between baseline recording and subsequent sessions at parietal site P6 only.

In a similar manner as described above, a 2 (hemisphere: left, right) × 4 (Session: one, two, three, four) repeated measures ANOVA was conducted to assess the effects of conditioning on liked brands across parietal sites. No significant effects were present across parietal electrode sites P5 or P6. Subsequent analysis via paired comparisons using a Bonferroni correction were then conducted at parietal sites during 200ms windows beginning at 400ms and ceasing at 1400ms at electrode sites P5 and P6. Whilst no effects were witnessed at site P5, results revealed that conditioning effects did occur, and were largest after one round of exposure at site P6 (*t* = −2.757; *df* = 19; *p* = 0.013, *d* = 0.51; two tailed; see Figure 6). This effect was seen to persist until 800ms (*t* = −2.455; *df* = 19; *p* = 0.024, *d* = 0.53; two tailed). To confirm the findings, further analysis of the event-related potentials (ERPs) using randomisation tests were conducted. Again, no effects were witnessed at site P5. In contrast, site P6 saw significantly more negative activity throughout baseline recordings than during subsequent sessions. This effect was seen to persist throughout the entire epoch, with greatest significance occurring between 400 and 800ms (*r* = 0.72, *p* = 0.001, two tailed; *r* = 0.73, *p* = 0.001, two tailed).

#### 3.3.2. Disliked Brands

**Figure 7:**
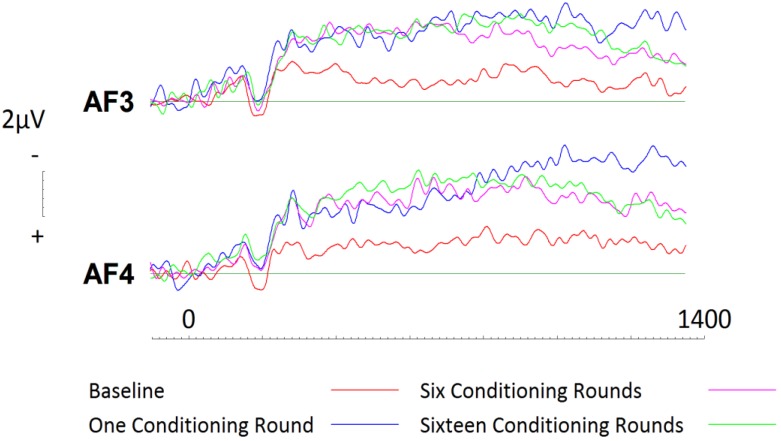
ERPs generated during each conditioning round for disliked brands across frontal electrode sites AF3 and AF4. Significant increases in activity were observed between session one and subsequent sessions as brands were conditioned more positively.

To assess conditioning effects with respect to disliked brands, a 2 (hemisphere: left, right) × 4 (Session: one, two, three, four) repeated measures ANOVA was conducted. Results revealed a significant effect of session (F(3,57) = 8.58, *p* < 0.001, η^2^ = 0.305). Paired comparisons using a Bonferroni correction were then conducted first for frontal sites. At left frontal site AF3, after one round of conditioning, activity was seen to increase significantly for approximately 400ms beginning at 400ms (see Figure 7; *t* = 4.136; *df* = 19; *p* = 0.001, *d* = 0.78; two tailed). Subsequently, randomisation tests were conducted (in the same manner as described above, for liked brands). The results revealed that baseline processing of brands was significantly more positive than subsequent sessions. Such effects were pronounced between 400ms and 1200ms, with greatest significance achieved between 800–1000 (*r* = 0.56, *p* = 0.001, two tailed). 

Across right frontal site, AF4, similar conditioning effects were witnessed (see Figure 7). After only a single round of conditioning, disliked brands were seen to elicit significantly greater activation between 400ms (*t* = 2.581; *df* = 19; *p* = 0.018, *d* = 0.53; two tailed) and 800ms (*t* = 3.208; *df* = 19; *p* = 0.005, *d* = 0.65; two tailed). Further analysis revealed that whilst subsequent conditioning maintained a change in activity across both frontal electrode sites, no significant increases or decreases were witnessed after the first round of conditioning. Randomisation tests were then conducted at site AF4 and yielded similar results, in that baseline recordings were significantly more positive than subsequent sessions. Whilst these effects persisted between 400 and 1000ms, greatest significance was achieved between 600 and 800ms (*r* = 0.60, *p* = 0.005, two tailed).

**Figure 8:**
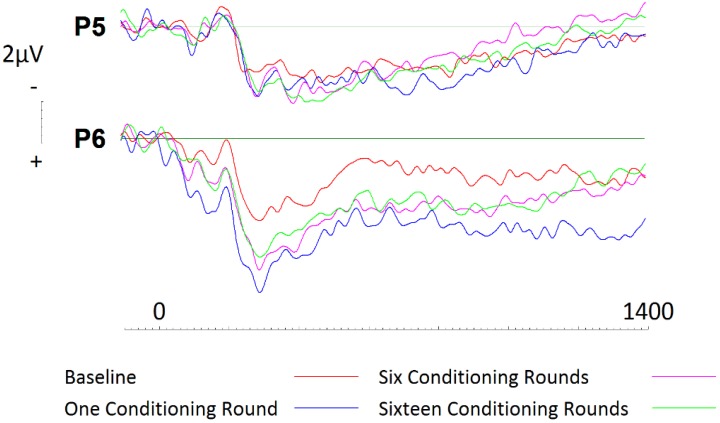
ERPs generated during each conditioning round for disliked brands across parietal electrode sites P5 and P6. Significant increase in cortical activity after only one round of conditioning for disliked brands across right parietal site P6.

In a similar manner as described above, a 2 (hemisphere: left, right) × 4 (Session: one, two, three, four) repeated measures ANOVA was conducted to assess conditioning effects across parietal sites P5 and P6. Whilst not significant, results related to *session* were seen to approach significance (*p* = 0.066). Additionally, pairwise comparisons using Bonferroni corrections and randomisation tests also revealed no conditioning effects across left hemisphere electrode site P5. In contrast, after only a single exposure to conditioning, disliked brand names were seen to elicit greater activity between 400ms (*t* = −3.946; *df* = 19; *p* = 0.001, *d* = 0.65; two tailed) and 1400ms (*t* = −3.175; *df* = 19; *p* = 0.005, *d* = 0.67; two tailed; see Figure 8) across right hemisphere electrode site P6. Furthermore, randomisation tests confirmed our significant findings and revealed significant differences between baseline activity and activity related to subsequent conditioning. Whilst the results were more conservative and revealed that baseline activity differed from activity elicited during subsequent conditioning rounds between 400 and 800ms (*r* = 0.73, *p* = 0.002, two tailed; *r* = 0.47, *p* = 0.04, two tailed), the effects were no less significant. 

#### 3.3.3. Frontal Asymmetry Effects

To assess asymmetry effects across right and left frontal electrode sites, 2 (Hemisphere: left, right) × 2 (Brand Type: liked, disliked) × 4 (Session: one, two, three, four) repeated measures ANOVAs were conducted for each 200ms block between 400ms and 1400ms. Throughout the entire epoch, a significant brand type by session interaction was present. This effect was most significant between 800 and 1000ms (F(1,19) = 7.00, *p* = 0.016, η^2^ = 0.269). Further investigation using pairwise comparisons revealed that liked brands were processed similarly across frontal electrode sites AF3 (M = −2.96) and AF4 (M = −2.90). In contrast, disliked brands were seen to elicit significantly greater activity across right hemisphere electrode site AF4 (M = −3.49) in comparison to left site AF3 (M = −1.73).

#### 3.3.4. LPP Effects

**Figure 9:**
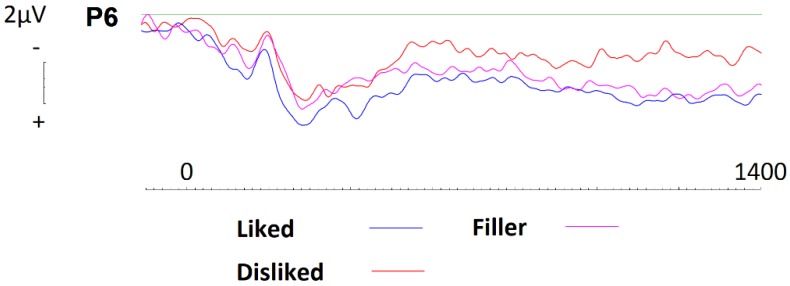
ERPs generated at site P6 during baseline recording for liked, disliked, and filler brands. Disliked brands were seen to elicit significant less activity than liked or neutral brands.

To investigate LPP effects across parietal sites P5 and P6, we conducted repeated measures ANOVAs with a single three-level factor (Brand type: liked, disliked, filler) between 400 and 700ms, 700 and 1000ms, and 1000 and 2000ms, in line with current literature [40]. A main effect of brand type was witnessed at baseline (F(2,38) = 5.70, *p* = 0.007, η^2^ = 0.231), whereby disliked brands (M = 1.80) were seen to elicit significantly less activity than liked (M = 2.47) or filler brands (M = 3.23). Whilst no significant effects were witnessed after 700ms, visual inspection of the ERPs suggest that an effect may be present (see Figure 9). Additionally, after having been exposed to conditioning, LPP effects cease. 

#### 3.3.5. Filler Brands

**Figure 10:**
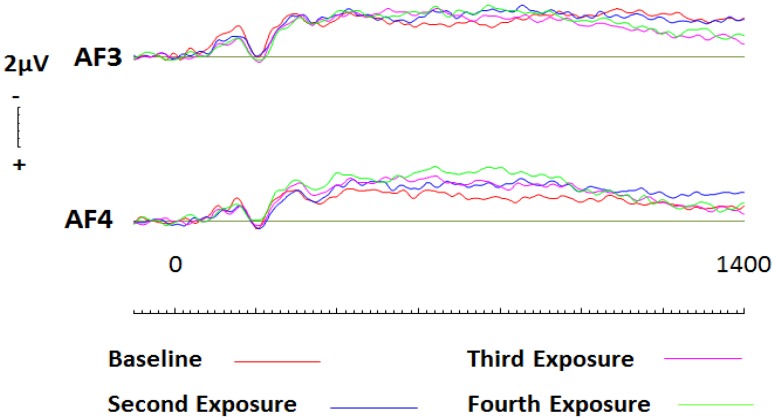
ERPs generated for all filler brands during each condition across frontal electrode sites AF3 and AF4. No changes between sessions were evident.

**Figure 11:**
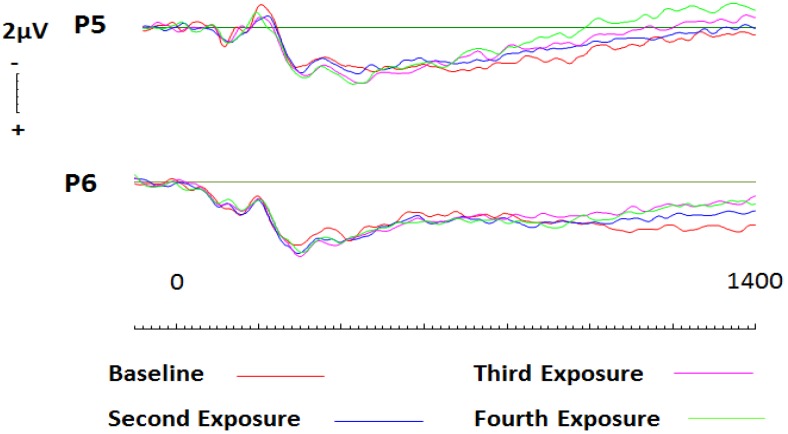
ERPs generated for all filler brands during each condition across parietal electrode sites P5 and P6. No changes between sessions were evident.

In a similar manner as the liked and disliked brands, 2 (Hemisphere: left, right) × 2 (Brand Type: liked, disliked) × 4 (Session: one, two, three, four) repeated measures ANOVAs were conducted for each 200ms block throughout the 1400ms epoch for filler brands at frontal and parietal sites. No significant effects were recorded for main effects or interactions for the filler brands at frontal (AF3 & AF4) and parietal sites (P5 & P6). Remember, filler brands were not conditioned, but repeatedly presented in exactly the same way as target brands. No session effects were evident across frontal sites (see Figure 10) or parietal sites (see Figure 11). Even though slight gradual increases in negativity can be seen across sessions, these effects are not statistically significant and thus, we can rule out any brand name repetition effects when looking at target brand conditioning effects.

## 4. Discussion

We used a multi-session, evaluative conditioning paradigm (varying numbers of conditioning rounds) with the aim of modifying participant’s attitudes towards their most liked and disliked brand names (established brand attitudes). Using a combination of traditional self-report measures as well as more innovative objective measures, we found differences in their ability to detect conditioning effects. Firstly, with regards to self-report, we found no conditioning effects. Secondly, even though there were some significant results those were inconsistent and cannot be used to neither support nor disprove our hypotheses. In contrast, EEG data suggest that not only is EEG capable of distinguishing between liked and disliked at baseline [38], but after subsequent conditioning it is also sensitive to varying numbers of conditioning rounds. Additionally, strengthening the finding of EEG as sensitive to evaluative conditioning, are the findings relating to the filler brands (those which were also repeatedly presented, but without any conditioning between the sessions), which revealed no session effects at all. This null finding strengthens the target brand ERP findings showing significant conditioning effects.

### 4.1. Self-Report and IAT

Analysis of self-report data (explicit rating performance) as well as IAT data, revealed no evaluative conditioning effects. As mentioned above, there were some significant IAT effects, but hose cannot be used to prove or disprove our hypotheses. It is widely accepted that self-report measures provide only a limited insight into consumer attitudes [53,54,55]. Our data mimic these findings and thus, represent strong and robust support with respect to existing literature. As already mentioned in the introduction section, it is suggested that the null findings pertaining to well established brands may arise as a result of cognitive pollution, an issue first raised by Walla and colleagues [29]. As participants were exposed to the conditioning paradigm, it may have been the case that this procedure exacerbated cognitive pollution, thus maintaining a lack of findings demonstrating evaluative conditioning effects.

Although IAT and self-report measures appear to be capable of distinguishing between liked and disliked brand names at baseline and after subsequent conditioning, our results indicate that they are incapable of detecting changes in strong and established brand attitudes as a result of evaluative conditioning. In fact, our results revealed that reaction times increased as participants were exposed to further conditioning. These findings support the idea that cognitive pollution may have increased as a consequence of the conditioning paradigm. Although it was unexpected, these findings seem to reiterate the findings of existing literature. In several studies, it has been suggested that the IAT may not exclusively measure implicit attitudes but instead, be influenced by cognition. It has been reported that mere supposition has resulted in IAT effects [1,56]. In cases where participants are instructed to think about the stimuli in either a positive or negative manner, the reaction times towards such stimuli are reported to have changed. As a result of such findings, it has been proposed that the IAT may not exclusively measure implicit attitudes [35,50] but instead, that it may reflect the ease of which participants are able to associate two categories of stimuli [57].

### 4.2. Event Related Potentials

Of all the measures utilized within the current study, ERPs arguably provide the greatest insight into the processing of brands, in particular attitude changes related to established brand names. To begin, our findings show that activity across left electrode sites did not differ for liked or disliked brands. This finding is not in line with current literature, which suggests that the processing of pleasant/approach related stimuli evoke larger potentials across left frontal electrode sites [58,59,60]. However, in contrast, across right hemisphere electrode sites, disliked brands were seen to elicit more negative brain activity. Whilst this finding adds support to current literature, which suggests unpleasant/avoidant stimuli result in an increase in right anterior activity, the combination of the above findings related to the asymmetry model reinforce the notion that firstly, liked brands are more resistant to the effects of conditioning than disliked brands, and secondly, that brand names may not be as inherently affective as other stimuli (i.e., stereotypes and out-group prejudices) utilized within the majority of psychological studies [34]. 

In addition to asymmetry effects, the results relating to the LPP provide further insight into the implicit processing and perceptions of brands. Interestingly, our study revealed that disliked brands elicited significantly less LPP activity than liked and filler (neutral) brands. This is in contrast to the majority of the literature that suggests LPP effects should be enhanced for both pleasant and unpleasant stimuli [40,41,61]. If our findings reflected those presented within existing literature, the filler brands in our study should have elicited the largest LPP effects given that liked and disliked brands were matched in terms of valence. It is possibly the case that our study provides support for the notion that brands, whether they be liked, disliked, or neutral, are not considered, at an implicit level, to differ in terms of motivational significance, which is the discussed psychological construct related to the LPP. Generally, larger LPP effects are evoked by evaluatively inconsistent stimuli (e.g., an unpleasant stimulus presented within a block of pleasant stimuli) than by evaluatively consistent stimuli (e.g., a pleasant stimulus presented within a block of pleasant stimuli [41,61]. Our data may suggest that disliked brands are less intrinsically significant. Additionally, our findings may also provide support for the conditioning effects witnessed throughout the study. Whilst one might expect disliked brands to have continued to be less motivationally significant than liked or neutral brands, conditioning appeared to distinguish any LPP effects. In sum, the findings related to LPP may suggest that attitudes towards disliked brands are more easily manipulated than are those regarding liked or neutral brands. 

In addition to the LPP effects published within this paper, were conditioning effects. Unfortunately, the majority of conditioning literature is yet to embrace implicit measures, let alone neurophysiological measures, which further adds to the scarcity of comparable research papers. Of the research available that utilises implicit measures, the majority fail to report changes in brand attitude. For instance, Shimp, Stuart and Engle, using the IAT, found that attitudes towards novel stimuli could be changed through the use of a conditioning paradigm however, conditioning attitudes for mature brands (i.e., Coke and Pepsi) was unsuccessful [14]. In addition, Gibson suggested that participants who had strong attitudes towards Coke and Pepsi did not show changes in attitude toward either of the two brands after having been exposed to conditioning [25]. In contrast to these studies, the present research highlights the need for more sensitive measures of implicit attitude. After only a single round of conditioning, the processing of liked and disliked brands was seen elicit differences in cortical activity. Moreover, after only a single round of conditioning, the largest increases in activity were recorded and conditioning effects for disliked brands continued to be presented after 16 rounds of exposure. 

Probably the most convincing piece of evidence to reinforce that the effects of conditioning witnessed within this paper were actually a result of conditioning, come from the filler brands. Although no significant changes in activity arose at either anterior electrode site, visual inspection would somewhat promote the notion that electrode site AF4 saw an effect. We understand this to be a result of "repetition effects" since no conditioning was undertaken, however the brands were repeatedly shown. It is possible that this repetition effect was also seen throughout the data for liked and disliked brands, thus enhancing the conditioning effects that were recorded. However, in contrast, at parietal site P6, no such gradual effects were seen for filler brands. With this in mind, it is assumed that the conditioning effects recorded at P6 for disliked brands are true conditioning effects and not a result of repeated brand presentations.

## 5. Conclusions

The current research adds to the vast amount of research that promotes the inclusion of implicit measures to assess brand attitude with a strong emphasis on EEG. Had EEG not been included in the present study, the effects of conditioning would have been completely absent. Through the inclusion of recording brain potential changes, we propose that the effects of evaluative conditioning occur at different levels of information processing and it appears as though traditional approaches lack the required sensitivity to competently and accurately assess consumer attitudes to this extent. 

In an applied sense, our results reveal exciting findings that suggest advertisers should not run the same ad for too long. The ERPs presented within the current paper indicated that although a significant degree of evaluative conditioning occurred after all three CS:US exposures, conditioning effects were most prominent after only a single pairing. This finding supports those presented by several authors, who report that a single conditioning trial will elicit a large effect, whereas subsequent conditioning trials will elicit smaller effects until a maximum is reached [6,18,62]. Given that the majority of the abovementioned authors relied on fictitious brands, more research in this area needs to be considered before any more robust conclusions can be drawn. 

Not only does extant literature suggest that conditioning effects diminish, but it has also been reported that over exposure to a single ad can in fact result in negative conditioning effects [63]. Furthermore, Sweldens, van Osselaer and Janiszewski report that different conditioning procedures might encourage different learning processes [20]. With this in mind, businesses may gain benefit from utilising different mediums when creating ad campaigns. Although this paper only focused on sounds as the conditioning medium, visual stimuli [64,65], olfactory stimuli [66], and gustatory stimuli [67] have all been used to condition positive affect. In principle, these current results emphasise the need of marketers to move away from traditional self-report measures and begin to address the issue explicit types of measures inherently possess.

In addition to the abovementioned findings, our results promote the idea that liked brands may be more resistant to the effects of evaluative conditioning. Although not directly linked to our study, the use of shock advertising may provide some support for this theory. Unlike typical advertising techniques, shock advertising aims at deliberately startling and offending its audience [68,69]. Although one would assume such advertising techniques would deter consumers from engaging with products, research has suggested that shock advertising can have positive effects on attention, memory, and behaviour [70]. Given our ERP findings that suggest the robust nature of well-established liked brands, it is suggested that businesses possessing such brands may be able to employ more controversial forms of marketing techniques without risking damage to their brands. 

In sum, further research within this area should focus on existing brands that are well-known to participants. This method will allow for the most comprehensive understanding of how brand attitudes are formed and modified. It may also be important to note that businesses may benefit from not rely on a single ad campaign for extended periods, but instead change campaign and consider focusing on different mediums by which to present their brands.
